# Disappearance of aura symptoms in patients with hemiplegic migraine after patent foramen ovale closure: a case report and literature review

**DOI:** 10.3389/fneur.2023.1267100

**Published:** 2023-10-12

**Authors:** Pian Wang, Fengyou Yao, Hongbo Zhang, Qian Yu, Yan Wang

**Affiliations:** ^1^ Department of Neurology, Chengdu Fifth People’s Hospital, Chengdu, China; ^2^ Department of Cardiology, Chengdu Fifth People’s Hospital, Chengdu, China

**Keywords:** sporadic hemiplegic migraine, whole-exome sequencing, aura symptoms, patent foramen ovale, headache

## Abstract

Hemiplegic migraine (HM) can cause significant functional impairment and negatively affect the quality of life of affected individuals. Emerging evidence suggests an association between migraines and congenital patent foramen ovale (PFO), which is a small opening between the atria of the heart that normally closes shortly after birth. This report describes a 34 years-old woman with sporadic hemiplegic migraine (SHM) who was diagnosed with PFO. Following percutaneous PFO closure, her hemiplegic symptoms disappeared, but her headache exacerbated. After 3 years of follow-up, her headache severity gradually reduced, and the frequency remained consistent at 2–3 times per year with no aura symptoms. This case highlights the dissociation between the resolution of hemiplegic symptoms and the persistence of headaches after PFO closure in sporadic HM. Patients with HM may experience changes in aura symptoms and headache severity after PFO closure. Before performing PFO closure in patients with hemiplegic migraine, the indications should be thoroughly understood.

## 1. Introduction

Hemiplegic migraine (HM) is a rare subtype of migraine with aura (MA) characterised by recurrent attacks of reversible motor weakness or paralysis (hemiplegia) on one side of the body (1). These attacks typically last from minutes to hours and are often accompanied by other migraine symptoms such as headaches, visual disturbances, and sensory changes (2). Sporadic HM (SHM) is a subtype of HM, and its exact prevalence is unknown; it is estimated to affect <0.01% of the population (3). The disease burden of SHM can be significant because of the frequency and severity of attacks. Hemiplegic episodes can cause significant functional impairment and negatively affect the quality of life of affected individuals (4).

Emerging evidence suggests an association between migraines and congenital patent foramen ovale (PFO) (5), which is a small opening between the atria of the heart that normally close shortly after birth (6). PFO allows the passage of small blood clots or other substances from the venous to the arterial circulation, potentially triggering migraines or stroke-like symptoms. A few case reports suggest that patients with HM may have co-existing PFO and that the patient’s clinical symptoms are alleviated after percutaneous PFO closure (7–10). Here, we report a case of SHM in a patient with a PFO. After percutaneous PFO closure, the hemiplegic symptoms disappeared, but the headache persisted.

## 2. Case presentation

A 34 years-old female patient began experiencing headaches at around age 6, which occurred 2–3 times per year. No apparent triggers were identified. Prior to each attack, the patient noticed neck stiffness. After noting Z-shaped flashing lights in her vision for about 10 min, she would develop a moderate throbbing headache (visual analogue scale [VAS] score of 5) on the right temporal and frontal areas, usually lasting for about 8 h and accompanied by nausea, vomiting, phonophobia, and dizziness. Resting in a quiet environment or consuming ibuprofen relieved these symptoms. After age 30, she started experiencing right-sided hemiparesis half an hour before the onset of headaches, which lasted for 2 weeks and occurred 2–3 times per year. Her migraine disability assessment questionnaire and headache impact test scores were 2 and 41, respectively. She was engaged in physical labour, denied smoking or alcohol consumption, and had no history of chronic or infectious diseases. Her mother died suddenly at age 66, and her father had bronchitis. She had six sisters and four brothers, and none had a history of similar episodes.

**Figure 1:**
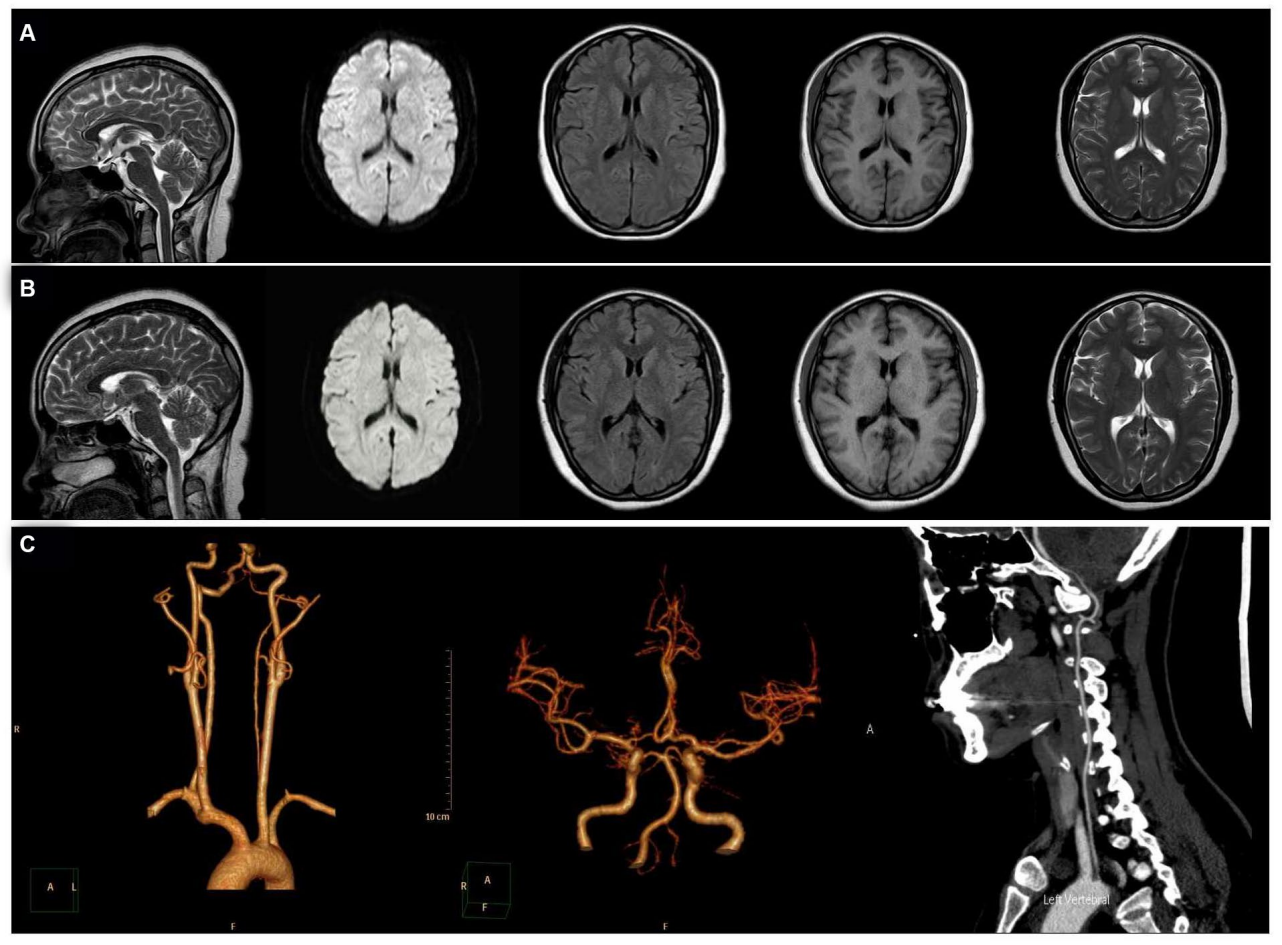
Magnetic resonance imaging (MRI). From left to right: sagittal, diffusion-weighted, fluid-attenuated inversion recovery, T1, and T2 sequences. **(A)** MRI showed no significant abnormalities during headache attacks with hemiparesis. **(B)** MRI showed no significant abnormalities at 6 months after patent foramen ovale closure. **(C)** Computed tomography angiography revealed left vertebral artery hypoplasia.

**Figure 2:**
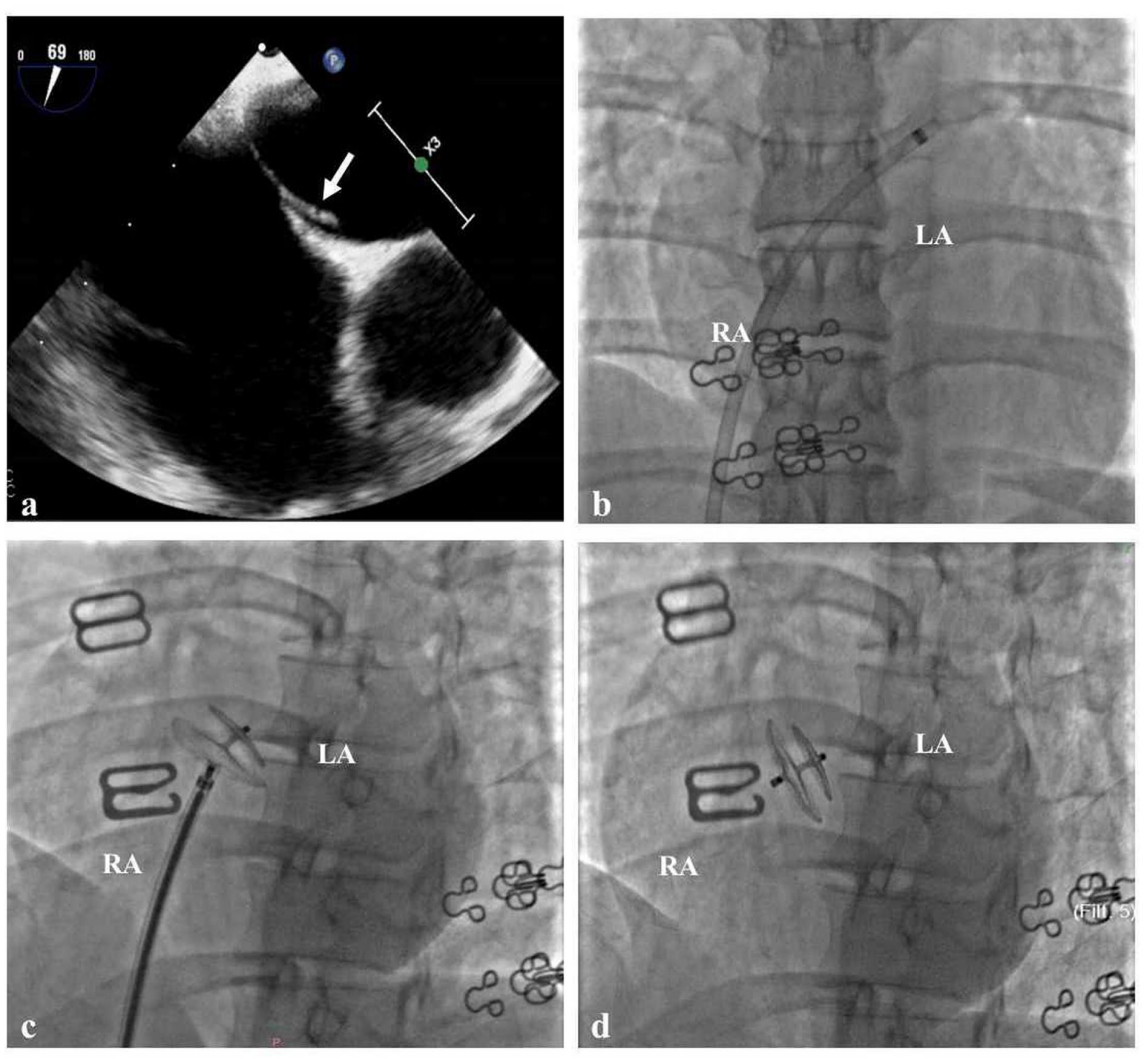
Cardiac Imaging in the Patient **(A)** Transoesophageal echocardiography showed that the patent foramen ovale was long tunnel-like (white arrow). **(B)** The delivery sheath passes through the PFO from the right to the left atrium. **(C)** The traction test of PFO closure was successful. **(D)** Made occluder successfully released.

When she was 34 years old, she was readmitted to the hospital because of right-sided limb weakness and a headache. A brain magnetic resonance imaging (MRI) scan during headache attacks with hemiparesis (Figure 1A) revealed no significant abnormalities. Computed tomography angiography revealed left vertebral artery hypoplasia (Figure 1C). During hospitalisation, a PFO with right-to-left shunting was observed. Transoesophageal echocardiography showed that her PFO was “long tunnel”-like (Figure 2A), which carried a higher risk of thromboembolic events. Blood tests, liver and kidney function tests, thyroid function tests, coagulation studies, and immunological examinations revealed no significant abnormalities. After evaluation by the cardiovascular department, she underwent transcatheter PFO closure according to the guidelines (11) and was discharged 2 weeks later (Figures 2B–D).

**Figure 3:**
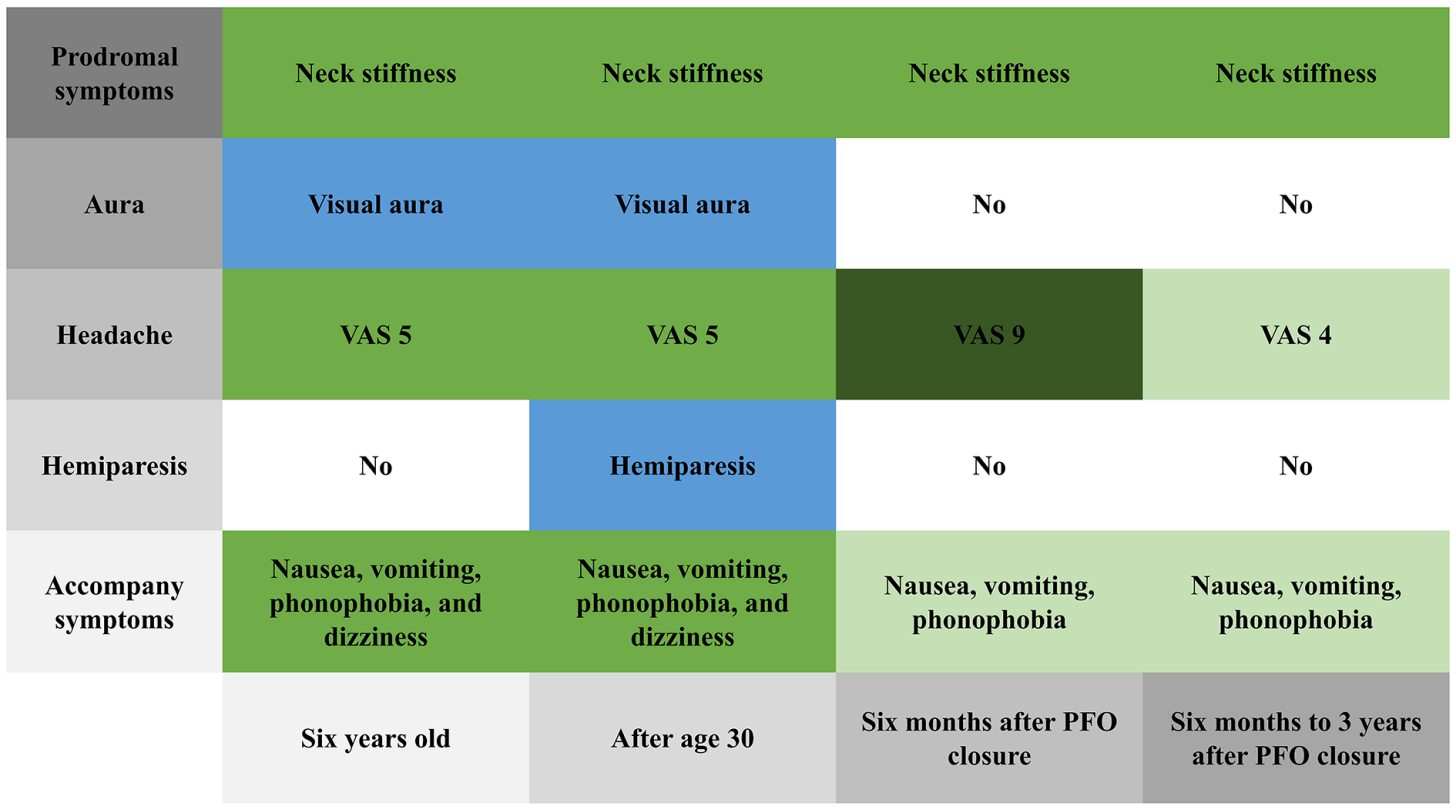
Changes in prodromal symptoms, aura, headache severity, hemiparesis, and accompanying symptoms from 6 years old to the 3 years follow-up after patent foramen ovale closure.

After she was discharged from the hospital, we followed her for 3 years. Six months after PFO closure, her headache severity worsened (VAS score 9) and she experienced a migraine attack lasting for 8 h, but without visual aura or hemiplegia. Her brain MRI was also normal (Figure 1B). Three years after PFO closure, she experiences a throbbing headache (VAS score 4) at the right temporal and frontal areas 2–3 times per year, with each attack lasting about 8 h. She had not experienced any more right-sided limb weakness; the Z-shaped flashing lights in her vision no longer occurred before headaches; and the accompanying dizziness during headaches had disappeared. Figure 3 shows the changes in prodromal symptoms, aura, headache severity, hemiparesis, and accompanying symptoms from the age of 6 years to the 3 years follow-up after PFO closure. With her informed consent, whole-exome sequencing was performed, and no genetic abnormalities were found.

## 3. Discussion

We report a case of migraine in a patient with PFO who experienced relief from aura symptoms after treatment. In this case, we considered the clinical diagnosis of HM valid. According to the International Classification of Headache Disorders 3rd Edition (ICHD-3) criteria (12), HM is a specific subtype of MA. The patient had a typical visual aura preceding each headache episode from age 6–30 years, which lasted for approximately 10 min. During this period, we diagnosed her with MA. Between the ages of 30 and 34, the patient also experienced right-sided limb weakness, occurring 30 min before each headache and lasting for approximately 2 weeks, which resolved completely after the attack. A brain MRI performed during headache episodes accompanied by hemiplegia did not reveal any significant abnormalities. Therefore, we revised the diagnosis to HM. According to the ICHD-3 description, hemiplegic symptoms can persist for several weeks (12), as in the present case. Finally, the patient underwent whole-exome sequencing, which did not reveal any pathogenic gene mutations associated with familial hemiplegic migraines. Therefore, we confirmed the diagnosis of SHM.

PFO is a residual opening between the septa primum and secundum that allows blood to flow from the right to the left atrium (13). Approximately 40–60% of individuals with MA have PFO, compared to 20–30% of the general adult population (14, 15). Although previous studies have demonstrated the impact of PFO on migraines, a definitive conclusion regarding the association between PFO and migraines has not been reached (16). HM has been sporadically reported in case studies on the relationship between HM and PFO closure (12). Table 1 summarises the cases of HM treated with PFO closure, showing partial or complete relief of symptoms in patients after closure. Interestingly, all previous case reports involved male patients. However, as this is a rare condition, sporadic case reports cannot be used to make inferences about the general population.

**Table 1:** Cases of hemiplegic migraine treated with patent foramen ovale closure.

Case report	Hemiplegic age	Gender	Aura symptoms	Diagnosis	Genetic tests	Radiological examinations	Heart exam findings	Prognosis after PFO closure
Brighina et al. (8)	30	Male	Basilar aura and left hemiplegia for 3 weeks	Atypical SHM	NA	Brain MR imaging was normal and MRA demonstrated hypoplasia of the basilar artery	PFO with a large right-to-left shunt.	Four year persisting remission of migraine
Perrotta et al. (9)	9	Male	Visual aura, paresis and paresthesia	FHM	ATP1A2	Brain MR imaging was normal	Large PFO and atrial septal aneurysm	Sudden improvement of the frequency of HM attacks
Lemka et al. (7)	14	Male	Visual aura, dysarthria, left hemiplegia, facial asymmetry	SHM	Normal	MRI + MRA imaging was normal	PFO with left-to-right shunt	Been free of SHM attacks for 3 years
Hu et al. (10)	Since childhood	Male	Visual aura, hemiparesis and hemianesthesia	HM	Normal	Brain MRI showed cortical necrosis and MRA demonstrated vasodilation of the branches of the left MCA and PCA	PFO	Substantially reduced frequency of episodes

HM, hemiplegic migraine; PFO, patent foramen ovale; NA, not available; MRA, magnetic resonance angiography; FHM, familial hemiplegic migraine; SHM, sporadic hemiplegic migraine; MCA, middle cerebral artery; PCA, posterior cerebral artery.

The exact pathogenesis of MA is still unclear. One proposed mechanism is cortical spreading depression (CSD), which is a transient wave of depolarisation originating from neurons and glia in the occipital cortex that slowly self-propagates in the cerebral cortex, followed by long-term inhibition of brain activity (17). However, the mechanisms by which CSD initiates, propagates, and triggers MA symptoms are undetermined. PFO may trigger migraine attacks by facilitating a microembolism, which activates the trigeminovascular system and leads to CSD (16). Genetic factors are also believed to play a role in the development of MA (18). Mutations in genes involved in ion-channel function, neurotransmitter release, and cortical excitability have been identified in some individuals with this condition (19). Our patient did not have any genetic mutations identified by whole-exome sequencing. Interestingly, as no further transient hemiparesis or other auras were observed after PFO closure, this clinical manifestation suggests that PFO may activate the trigeminovascular system and can lead to CSD by facilitating a microembolism.

Kato et al. (20) conducted a retrospective cohort study evaluating the effect of atrial septal defect (ASD) closure on migraine attacks and found that 11 of the 29 patients who had HM prior to ASD closure reported exacerbations of migraine attacks, but most of them improved after a median of 2.5 months post-surgery. Yankovsky and Kuritzky reported a 48 years-old male with migraines with sensory and visual aura who experienced daily attacks for 6 months the day after undergoing percutaneous ASD closure (21). Riederer et al. reported a 27 years-old female with MA who experienced recurrent migraine attacks almost daily after percutaneous ASD closure (22). Six months after PFO closure, headache severity worsened in our patient. Platelet activation on the surface of the implanted device and subsequent secretion of serotonin by activated platelets have been proposed as one potential mechanism for headache exacerbation (20). Implanted devices or tensile deformation of the interatrial septum may induce the release of atrial natriuretic peptide, which is associated with migraine attacks (21, 22).

In our case, the patient presented with hypoplasia of the left vertebral artery. Autopsy and angiography studies have reported that the frequency of this congenital variation ranges from 2 to 6% (23). Brighina et al. described a patient with atypical SHM associated with a PFO and basilar artery hypoplasia (8). The relationship between anatomical variations in blood vessels and hemiplegic migraines remains under investigation. MA was found to be associated with a perfusion deficit not limited to a specific vascular territory by Förster et al. (24). In previous studies, up to 23% of patients with HM were found to have white-matter signal abnormalities on T2 sequences outside migraine attacks (25, 26). Additionally, cortical oedema has been documented during HM attacks, both in FHM (26, 27) and SHM (28, 29). However, no brain MRI abnormalities were found in our case.

MIST (Migraine Intervention with STARFlex Technology) (30), PRIMA (Percutaneous Closure of PFO in MA) (31), and PREMIUM (Prospective, Randomized Investigation to Evaluate Incidence of Headache Reduction in Subjects With Migraine and PFO Using the Amplatzer PFO Occluder to Medical Management) (32) are three randomized controlled trials that investigated the efficacy of PFO closure in patients with migraine. Although a meta-analysis of these studies indicated that PFO closure may offer benefits in reducing migraine attacks and migraine days (5, 33–36), there is still no good-quality evidence to support PFO closure reducing the intensity and severity of migraine headaches (37). From our perspective, based on the safety of PFO closure, relieving migraines should be regarded as an additional benefit rather than a primary goal. These interventions reduce the risk of stroke or myocardial infarction resulting from paradoxical embolism, and preventing systemic embolic events is a lifelong endeavour (38).

## 4. Conclusion

This case highlights the dissociation between the resolution of MA and the persistence of headaches after percutaneous PFO closure in a patient with SHM. There is still a lack of effective evidence that PFO closure is beneficial for the prevention of HM attacks. Patients with HM may experience changes in aura symptoms and headache severity after PFO closure. Before performing PFO closure on patients with HM, the indications should be thoroughly understood.

## Data availability statement

The original contributions presented in the study are included in the article/supplementary material, further inquiries can be directed to the corresponding author.

## Ethics statement

Written informed consent was obtained from the individual(s) for the publication of any potentially identifiable images or data included in this article.

## Author contributions

PW: Supervision, Writing – original draft. FY: Writing – review & editing. HZ: Investigation, Writing – original draft. QY: Performed the PFO closure procedure and advised on the manuscript. YW: Writing – original draft.
